# Neighborhood-Based Social Capital and Depressive Symptoms among Adults: Evidence from Guangzhou, China

**DOI:** 10.3390/ijerph182111263

**Published:** 2021-10-27

**Authors:** Sanqin Mao, Jie Chen

**Affiliations:** 1 School of International and Public Affairs, Shanghai Jiao Tong University, Shanghai 200030, China; alicemao01@sjtu.edu.cn; 2 China Institute for Urban Governance, Shanghai Jiao Tong University, Shanghai 200030, China

**Keywords:** social capital, social participation, volunteering, urban China, urban governance

## Abstract

This study examined the association between neighborhood-based social capital (NSC) and depressive symptoms in the context of urban neighborhoods in China, with special attention given to the association heterogeneity across socioeconomic groups. Drawing on cross-sectional data collected from 39 neighborhoods in Guangzhou, this research demonstrated that adults’ depressive symptoms were higher among those with lower cognitive (trustworthiness, reciprocity, and cohesion within a neighborhood) and structural (social network and participation) dimensions of NSC. Further analysis showed that the negative association between NSC and depressive symptoms was significantly heterogeneous across socioeconomic groups. Specifically, this negative relationship was more prominent in the lower socioeconomic classes than in the upper socioeconomic classes, indicating that the lower accumulation of NSC among disadvantaged groups may aggravate depression unequally across social classes. In addition, the negative association between social participation and depressive symptoms was stronger for people who are older or unemployed. The findings of this study not only provide new evidence concerning the significance of the beneficial effects of NSC in the Chinese context, but also, more importantly, highlight that NSC plays a crucial role in creating mental health inequality across social classes. Thus, the relevant social interventions including fostering neighborhood relationships and social activities should be carefully tailored against the backdrop of community building during the urbanization process. The implications of our study for urban governance to promote healthy cities are discussed.

## 1. Introduction

Depression has increasingly become a worldwide mental health concern. It has been reported that depression affects 120 million people worldwide [1], and it ranks 15th among the leading contributors to disability-adjusted life years (DALY) [2]. Depression not only adversely affects normal life but also substantially raises the risk of various physical illness, such as addiction, stroke, self-injury, and cardiovascular disorders [3,4,5,6]. In order to reduce the occurrence of depression, a growing number of studies have tried to examine the potential contributors to depression. A large body of research has found that depressive symptoms vary across different population groups, such as age cohorts [7,8], gender groups [9], and migration categories [10,11]. Research has also revealed that neighborhood characteristics, such as the neighborhood’s physical environment (e.g., built environment, green spaces, and communal spaces) [1,7,10], social environment, including relationships among neighbors (e.g., trustworthiness, social cohesion, and shared norms of reciprocity) [4,12,13,14], and participation in neighborhood activities (such as volunteering and participation in associations) [8,13,14], have specific links to depressive symptoms. 

While a plethora of studies have delved into the linkage between neighborhoods’ social environment and mental health in developed countries, intellectual inquiries about how this link works in developing countries has only just begun in recent years [13]. It has been reported that people from developing countries are more likely to be depressed than ever before, and depression has become one of the most important risk factors for disability and morbidity [15,16]. However, little is known about the social environment drivers of depression in these populations. To that end, there is an urgent need to disentangle the mental health benefits of residential social environments in developing countries for future public policies and social interventions. This study contributes to the literature by examining the association between neighborhood-based social capital (hereafter NSC) and depressive symptoms in the Chinese context, where the rates of depressive symptoms and depression have increased rapidly, and have topped 37.9% and 4.1%, respectively [17]. To date, only a few studies have examined the link between NSC and depressive symptoms in Chinese cities [13,18,19]. However, these studies have only focused on people who are older. Furthermore, studies concerned with the association of NSC with depressive symptoms have been left with many questions, considering that social capital is a multi-dimensional concept, and can take various forms. More studies are needed not only to investigate the relationship between NSC and depressive symptoms across population groups of different age cohorts, but also on whether and to what extent different forms of social capital are associated with depression. 

Using data from a city-wide survey of 39 neighborhoods completed in early 2013 in Guangzhou, this study attempts to investigate the association between two forms of NSC and depressive symptoms, and further investigate whether this association may vary across socioeconomic groups. This question is of great significance for understanding the role of neighborhoods in preventing and treating mental illness within China’s phenomenal urban transformation. This study contributes to the literature in three aspects. First, it makes the first attempt to systematically explore the relationship between NSC and depressive symptoms among adults in the Chinese context. Second, it breaks down the notions of different forms of social capital, and distinguishes between the effects of different forms of NSC. Third, it contributes to the existing literature on depression and to the debate on the significance of neighborhoods by examining the heterogeneous relationship between NSC and depressive symptoms across socioeconomic groups.

In this study, we first review the urban sociology literature on the association of NSC with depressive symptoms, including works pertaining to Chinese cities, and formulate several hypotheses accordingly (Section 2). In Section 3, we present the methodology and data of the study. We then analyze the extent to which different forms of NSC are related to depressive symptoms, and further explore the heterogeneous relationship between NSC and depressive symptoms across socioeconomic groups (Section 4). In Section 5, we discuss implications, limitations, and avenues for future study. Finally, the main findings are highlighted in Section 6.

## 2. Literature Review and Hypothesis Development

### 2.1. Association between Neighborhood-Based Social Capital and Depression

Social capital has received much attention in the past decades since Putnam, in his seminal work, *Bowling Alone*, argued that the American society is facing the loss of social capital, which leads to a myriad of social problems [20,21]. Despite the growing number of studies on social capital, we still lack of a precise definition for the term. Existing studies have explored social capital from different sources (e.g., neighborhood, employment, and family social capital) and in different forms (e.g., bridging vs. bonding, structural vs. cognitive, trust vs. reciprocity, individual vs. collective) [10,11,19,22,23]. This study focused on individual-level neighborhood-based social capital, which is one of the most important sources of social capital among adults. It adapts the typical typology of social capital to distinguish between two forms of NSC: neighborhood-based cognitive social capital (hereafter NCSC), and neighborhood-based structural social capital (hereafter NSSC) [19,20,21,24]. NCSC refers to residents’ subjective perceptions and evaluations of social relationships within local neighborhoods, such as perceptions of the trustworthiness of neighbors, solidarity among neighbors, shared norms of reciprocity, and commitment within the neighborhood [10,11,14]. Unlike NCSC, NSSC is objective and measurable, and reflects residents’ social behavior in local neighborhoods. Common indicators of NSSC include neighborhood acquaintances, neighbor interactions, and neighborhood participation (primarily social activities and volunteering) [10,14,25]. 

Previous studies have revealed that NCSC has more powerful protective effects against depressive symptoms than NSSC [24,26]. Residents who perceive their neighborhood to have a high level of mutual trust [27], social cohesion [14,23], and reciprocity [28] were significantly less likely to suffer from depressive symptoms in Western contexts. The main underlying mechanisms are that trust, reciprocity, shared norms, and values within a neighborhood can promote personal information exchange and knowledge transmission, affect the allocation and utilization of resources and services, back up the social credibility of residents, support healthy behaviors, and offer social support [27,28,29,30]. As for NSSC, current studies have revealed that social networks and neighborhood participation are significantly negatively associated with depressive symptoms [31,32,33]. Neighborhood participation, which is also a multidimensional construct in the literature, covers a wide range of activities, such as political, social, leisure, and cultural activities in the neighborhood. Several explanations for the obvious negative link between neighborhood participation and mental health have been proposed. For a start, participation in neighborhood activities provides individuals with opportunities to share information and exchange opinions mutually, which helps people to decrease feelings of loneliness, and to embrace healthier habits and lifestyles [33,34]. Furthermore, joint activities increase social contact, which can foster supportive and caring relationships, and consolidate social integration [8]. People can receive assistance and support more easily through these relationships, thereby reducing the risk of depression. Moreover, participation may make people recognize that they are part of the neighborhood, which promotes confidence, and inspires them to be more enthusiastic about neighborhood affairs [10]. 

Despite the strong evidence for the inverse relationship between NSC and depressive symptoms, some studies have revealed no significant relation between them [10,14,31]. A 2005 literature review [26] reported that, of the 11 works included in the study, seven revealed significant negative relationships between NCSC and depression, but only three revealed significant negative relationships between NSSC (primarily social engagement) and depression. Cao et al. [24] and Lu et al. [19] summarized the reasons behind these inconclusive findings regarding NSC and depression. First, due to differences in the measures and conceptualization, it is difficult to compare results across relevant works. Second, many studies have only focused on one component of NSC, but have not included measures of both NCSC and NSSC. These issues within the literature pinpoint that further study is needed, and that both levels of measures should be included. 

### 2.2. NSC and Its Association with Depression under China’s Unprecedented Urban Spatial Restructuring 

Compared with the large volume of empirical evidence focused on Western countries, fewer cases have been located in the context of urban China, where the phenomenal urban transformation has brought about fundamental changes in neighborhoods’ physical and social environments during the recent decades [35]. In China, the 40-plus years of reform and opening up have radically changed people’s lives, including neighborhood life [36]. The 1998 reform, in particular, aimed to end the welfare allocation of housing, with a view to developing urban real estate as a growth anchor [35,37]. Since then, phenomenal urban expansion into the surrounding countryside and large-scale inner-city regeneration have continually reshaped urban spatial and social structures. In almost every major city, tract after tract of traditional inner-city neighborhoods have given way to high-rise office block towers, new commodity housing neighborhoods, and condominiums [36,37]. In sprawling suburbs, new commodity housing estates and development zones have replaced farmlands at amazing speed [37,38,39]. 

Underlying the unprecedented urban transformation is the increased rate of inter-city and intra-urban migration, which were inhibited before the reform. For example, it has been reported that around 10% of the citizens in Guangzhou have experienced at least one move per year from 2000 to 2012, and the city’s migrant population has increased from 2.99 million to 8.83 million from 2000 to 2020 [38,40,41]. The prevalent phenomenon of migration has created different imprints in a variety of neighborhoods, which constitute a mosaic of enclaves in Chinese cities [35,39]. Obviously, urban spatial developments and the associated population dynamics have impinged heavily on NSC, and would have undermined the long-established social networks. Unlike traditional compounds, where intensive interactions among peer-neighbors engendered the so-called *dayuan*, or big compound sub-culture, current residential compounds have transformed to a locale comprising strangers who do not readily trust each other [42]. It has been indicated in earlier studies that neighborhoods, especially newer neighborhoods, have seen a decline in social interactions [43,44]. The prevalence of individualization, the pluralization of lifestyles, and advances in online communication tools have further undermined neighborly connections [45]. Meanwhile, the management of the neighborhood rests upon the estate management firm, usually a subsidiary of the developer, which reduces the dependence on neighbors, hence decreasing the intensity of social interaction. In this context, some scholars have indicated that China is facing the demise of neighborhoods, and have shown empirical evidence on the side of the ‘community lost’ thesis during the urbanization process in the past few decades [46]. 

Although the reduction in the social capital of the neighborhoods has been explored, few have examined how the neighborhood’s social environment is related to depressive symptoms in Chinese cities. A small number of studies have begun to focus on the group of people who are older in China, and have explored the link between NSC and depressive symptoms in this group [18,19,24]. For example, Cao et al. [24] revealed that individual social networks, reciprocal exchange, and mutual trust among neighbors were negatively associated with depressive symptoms among older adults. Similarly, Wang et al. [18] found that reciprocal exchange was significantly related to a lower level of depression among older people. As for the effect of NSSC, Wang et al. [18] focused on a group of older people, and observed significant associations between neighborhood social participation and the risk of geriatric depression. Notably, the literature in the Chinese context has mainly focused on the group of people who are older. This study attempted to empirically analyze the link between NSC and depressive symptoms among adults based on a typology combining the cognitive and structural forms of social capital.

### 2.3. Hypothesis Development

Based on previous empirical studies, our model includes two main testable hypotheses:

**Hypothesis** **1** **(H1).** *neighborhood-based cognitive social capital—neighborhood trust, reciprocity and social cohesion—will be negatively associated with depressive symptoms, after controlling for socioeconomic variables.*

**Hypothesis** **2** **(H2).** *neighborhood-based structural social capital—networks and social participation—will be negatively associated with depressive symptoms, after controlling for socioeconomic variables.*

While, according Howley et al. [47], the neighborhood plays a significant role in some groups’ daily lives, NSC may have little or no impacts on other groups. Accordingly, we expect the association between NSC and depressive symptoms to differ among population groups. In particular, for residents who spend a considerable amount of their time in their residential neighborhood and are less affluent and have scant personal resources, which makes them reliant more on resources from their neighborhood (including the people who are older, unemployed, and of a lower socioeconomic status), a stronger relationship between NSC and depressive symptoms is expected. In contrast, residents who are more physically mobile and have much personal connection with social networks outside of the neighborhood are expected to be less likely to be influenced by NSC [48]. Thus, we suspect that the neighborhood may be particularly relevant for mental health in populations of people who are older or of lower socioeconomic status. However, there is high uncertainty regarding other socioeconomic factors such as gender, family structure, education level, and marital status. We did not plan to derive a specific hypothesis for the heterogeneous relationships between NSC and depressive symptoms, but have left them for the following empirical investigations.

## 3. Data and Research Method

### 3.1. Data Collection

In this research, we analyzed the relationship between NSC and depressive symptoms in the city of Guangzhou, China. Guangzhou is one of the largest cities in the south of China, with a resident population of over 18 million in 2020 [41]. Guangzhou has been considered as a forerunner in urban regeneration, but has been confronted by severe social problems, such as aging and residential segregation [39]. Consequently, the findings of this study have practical implications for policy-makers regarding the construction of healthy cities. The data were derived from a city-wide household survey conducted jointly by a collaboration of Sun Yat-sen University, Hong Kong Baptist University, and Duke University. Conducted from October 2012 to January 2013, this survey was a large-scale, face-to-face investigation on neighborhood-based social capital and neighborhood governance. To obtain a representative sample, the research team used a multistage stratified random sampling method to recruit respondents. The detailed design process was as follows: first, 30 streets (*jiedao*) or sub-districts were sampled from the list of all streets located within the city based on a GIS sampling method; second, two or three neighborhoods (*xiaoqus*) within the selected street were randomly sampled, based on the neighborhood size and location; third, within each neighborhood, respondents were selected based on the interval sampling method, and interviewed by trained interviewers. The exact number of target respondents from each neighborhood depended on the population size of the neighborhood and the addresses of the respondents. Respondents were approached personally by interviewers visiting them at home, and were asked to complete a questionnaire. In total, 39 neighborhoods that varied in location, type, and size were selected for this research. In total, 1771 respondents have successfully completed the survey. 

The age, gender, education, and *hukou* status ratios of participants were consistent with the 2010 Population Census of Guangzhou City. Specifically, people aged 20–64 account for 88.03% of the sample, and 81.91% of the entire population. In terms of gender, males constitute 44.32% of the sample, and 52.26% of the entire population. In terms of *hukou* status, the share of people with non-local *hukou* in the sample is 28.23%, whereas the share indicated by the population census is 36.32%. The relatively lower proportion of the migrant sample is possibly due to the informal housing neighborhoods such as urban villages and self-built settlements not being covered. Thus, the group of non-local *hukou* holders need to be considered in future research.

### 3.2. Measures

#### 3.2.1. Dependent Variable: Depressive Symptoms

Depressive symptoms in the week before the survey were measured by the 20-item Likert scale questionnaire of the CES-D 20 (Center for Epidemiologic Studies Depression Scale) [49]. This measurement instrument has been widely used and verified to have high reliability for adults in many countries [16,18]. Participants were asked to report the frequency of experiencing depressive symptoms such as feeling scared, feeling upset, and feeling lonely. Their answers to each item were rated on a four-point scale. A higher total score indicated greater levels of depressive symptoms. The total CES-D 20 scores were assessed (Cronbach’s alpha = 0.85) and used as the dependent variable. 

#### 3.2.2. Independent Variables: NCSC, NSSC and Socioeconomic Variables

NCSC, consisting of neighbor trustworthiness, reciprocity, and social cohesion (feelings of commitment and trust) dimensions [19,23,25,50], was calculated from six items (see Table 1). For all six statement, respondents’ responses were scored on a 5-point scale from 1 (strongly disagree) to 5 (strongly agree). According to our survey, about 53.28% of respondents agreed or strongly agreed that their neighbors are trustworthy, and 63.26% of respondents agreed or strongly agreed that people in the neighborhood are helpful. The average of the respondents’ answers to the six items was 3.59, between the choice of ‘neutral’ and ‘agree’. Considering multicollinearity of these dimensions, the method of principal component analysis was adopted. As shown in Table 1, the method indicated that one single component explained 66.73% of the total variance (Cronbach’s alpha = 0.85). Therefore, the overall factor score was used to represent NCSC.

NSSC was measured in two dimensions [14,24]. The first dimension was the discussion network, which was assessed by the logarithm of the number of neighbors with whom respondents could discuss private affairs. The second dimension was social participation. Previous empirical studies in Asian populations have reported that participation in welfare, volunteering, and leisure activities is significantly associated with lower levels of depressive symptoms [14,19,50]. Thus, social participation was measured by attendance at volunteer activities such as donating clothes, money or blood, and leisure activities such as walking, exercising, shopping, and other recreational activities in the communal space with neighbors in past six months. A binary variable was calculated, with respondents who do not take part in any kind of activity having a value of 0, and the rest having a value of 1. 

According to existing work on depression [14,19,24], the set of personal variables included age, gender, *hukou* status, presence of children, years of schooling, homeownership, length of residence, self-perceived socioeconomic status, and employment conditions (unemployed or not). 

### 3.3. Analytic Strategy

The analysis was based on the complete information provided by 1771 respondents. We began by providing the descriptive statistics of the respondents. The multicollinearity of the independent variables was then checked before running the models. After that, the regression analysis was applied to unravel the relationships between the two types of NSC and depressive symptoms. To further explore the heterogeneous relationship between NSC and depressive symptoms, interaction terms were then added. Only interaction terms which obviously enhanced the goodness of fit of the model were included. Furthermore, because previous studies have highlighted the significant role of neighborhoods in the life of people who are older, the interactions of people who are older and two types of NSC were added to capture the particular effect of NSC for the group of people who are older. 

## 4. Analysis Results

### 4.1. Descriptive Analysis

Table 2 shows the basic characteristics of the sample. The mean age of the respondents is approximately 45 years. More than half of the total sample is female. Around half of the respondents had completed higher levels of education after high school. As for *hukou* status, 71.77% of the sample have Guangzhou *hukou*. With respect to tenure status, near 80% of respondents are homeowners, reflecting the high rate of homeownership since 1998 [38]. In terms of occupation status, 7.45% were unemployed. Furthermore, 42.02% of participants consider themselves to be middle class or above, whereas 57.98% of participants define themselves as being part of the lower socioeconomic class. Referreing to NSSC, on average, residents have five neighbors with whom they can discuss private affairs. In the survey, 81.25% of the participants reported that they had engaged in at least one activity. On the whole, most respondents’ CES-D 20 scores were below 16 (the cut-off point of clinical depression), and the average CES-D 20 score of the total sample is 9.98, with a range from 0 to 44.

### 4.2. Model Results

The relationships between the two types of NSC and depressive symptoms are presented in Model 1 (see Table 3). The heterogeneous relationships between NSC and depressive symptoms are further explored in Model 2 and 3. The increased adjusted R^2^ indicates that Model 2 and Model 3 have a better predictive value than Model 1, and obtained satisfactory results. As shown in Model 1, the subjective socioeconomic status and employment status are key differentiators of depressive symptoms. This finding is in line with existing studies showing that people who are of a lower socioeconomic status (β = 1.334, *p* < 0.001) and people who are unemployed (β = 3.499, *p* < 0.001) are more likely to suffer depressive symptoms and report a high risk of mental illness [51,52]. 

#### 4.2.1. Association of Cognitive and Structural Social Capital with Depression

The NCSC (interpersonal trust and reciprocity in neighbors and neighborhood cohesion) was associated with lower risks of depressive symptoms, as shown in Model 1 (β = −0.963, *p* < 0.001), after controlling socioeconomic variables. This is consistent with previous studies in other countries and other studies from China that NCSC benefits to people’s mental health [13,19,24]. It is clear that living in a trusting, reciprocal, and close-knit neighborhood could reduce depressive symptoms by generating positive states and emotional support. As for NSSC, social network group (β = −1.176, *p* < 0.01) and social participation (β = −2.043, *p* < 0.001) were significantly and negatively associated with levels of depressive symptoms, which is consistent with previous research [13,53]. Resources embedded in social network and social participation, including volunteering, welfare activities, and leisure activities are beneficial to psychological well-being for dealing with life stressors. 

#### 4.2.2. Heterogeneity Analysis

As discussed in Section 2, the relationships between NSC and depressive symptoms may obscure various differences across different population groups. On that account, the details of the heterogeneity of the relationships between NSC and depressive symptoms were examined. There were significant differences in age, employment status, and subjective socioeconomic status as presented in Model 2 and 3. 

##### Age

**Figure 1:**
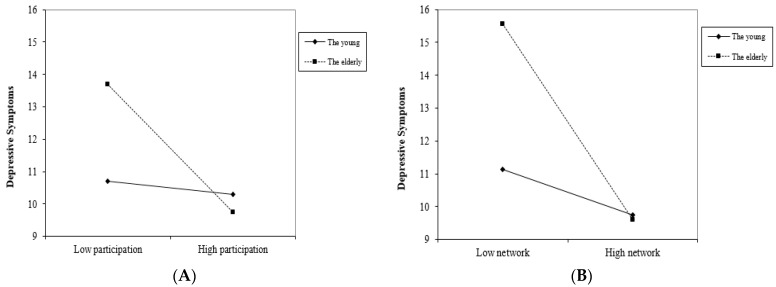
The relationship between NSC and depressive symptoms varied among age groups. (**A**) refers to the relationship between social participation and derpessive symptoms among age groups; (**B**) refers to the relationship between discussion network and depressive symptoms among age groups.

As shown in Model 3, the association between social networks with neighbors and depressive symptoms was more prominent in the older population than in the younger population (β = −1.363, *p* < 0.05). In addition, the significant link between social participation and depressive symptoms is accentuated for the older group (β = −5.616, *p* < 0.001). For ease of presentation, Figure 1 displays how the relationships between both neighborhood discussion networks and social participation, as well as depressive symptoms varied among age groups, after controlling for socioeconomic variables. As shown in Figure 1A, social participation was associated with lower levels of depressive symptoms, and this effect was more notable for people who are older. Similarly, Figure 1B suggested that the larger the neighborhood discussion network, the fewer the depressive symptoms. This effect was stronger in the older than the younger group.

##### Socioeconomic Status

**Figure 2:**
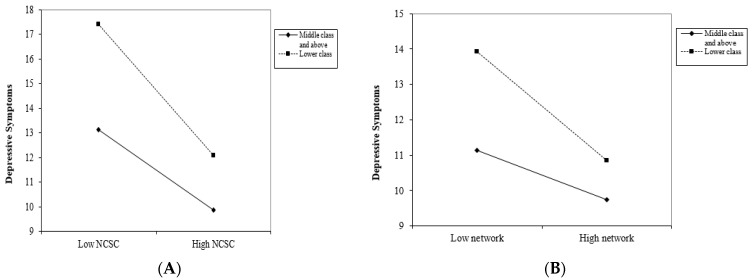
The relationship between discussion network and depressive symptoms varied among socioeconomic groups. (**A**) refers to the relationship between NCSC and derpessive symptoms among socioeconomic groups; (**B**) refers to the relationship between discussion network and depressive symptoms among socioeconomic groups.

There was a significant interaction effect between socioeconomic status and NCSC on depressive symptoms (β = −0.597, *p* < 0.01). This indicates that the negative association between NCSC and depressive symptoms was stronger in lower socioeconomic groups than higher socioeconomic groups. The interaction effect between socioeconomic status and neighborhood discussion networks (β = −1.497, *p* < 0.05) was also significant. As shown in Figure 2, despite trust, reciprocity, perception of social cohesion, and discussion networks being associated with fewer depressive symptoms for all classes, the effect was more prominent for individuals in a lower socioeconomic class than those in the middle class or above. 

##### Employment Status

**Figure 3:**
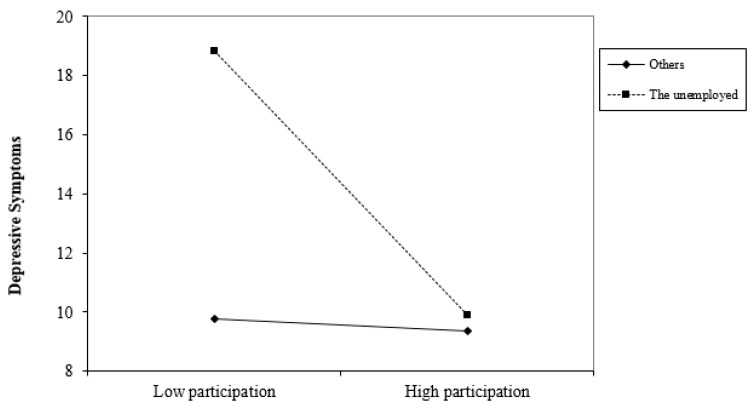
The relationship between neighborhood participation and depressive symptoms varied among people with different types of employment status.

Interaction analysis also showed that social participation was significantly more significant for people who are unemployed (β = −12.301, *p* < 0.001). This suggests that social participation may play an important role for those who have no work. Unemployment has been confirmed as a significant determinant of depression, mainly through threats to individual identity, and economic pressure [54]. As shown in Figure 3, neighborhood social participation may buffer the typically negative link between unemployment and depressive symptoms.

## 5. Discussion

In line with the findings revealed in developed countries, this study has confirmed the significant inverse association between NSC and depressive symptoms in China. Residents living in neighborhoods that are stronger in mutual trust, reciprocity, and social cohesion, along with those with larger discussion networks and higher levels of social engagement, were more likely to report fewer depressive symptoms than others. The result further revealed that the association between NSC and depressive symptoms was heterogeneous across population groups. Specifically, the association of NSSC (including neighborhood discussion networks and social engagement) was stronger in the older group than in the younger group. People who are older have limited mobility or are restricted in their means of transportation, and they cannot make or are unaccustomed to making use of social media connections. Accordingly, they tend to develop more neighborhood contacts and engage in neighborhood activities. In this context, neighborhood-based discussion networks and neighborhood activities are important for people who are older, as confirmed by a large body of research [13,55]. 

Our findings have also verified that the association between NSC and depressive symptoms is more prominent in the lower socioeconomic group than in higher socioeconomic groups. This pattern suggests that NSC is still a significant source of support for people who are of lower socioeconomic class. It is understandable that higher-class individuals generally tend to have larger social networks and more social support beyond the boundaries of the neighborhood, compared with individuals in a lower socioeconomic class [54]. Moreover, those in the lower class are more likely to be trapped in the neighborhood, and restricted to spending time in the neighborhood, whereas the upper class are more able to change residence for adjusting their housing consumption [38]. Previous studies have demonstrated that lower socioeconomic status can seriously threaten people’s psychiatric health [51,52]. Residents with the lowest incomes in a given location are 1.5 to 3 times as likely to suffer depression or anxiety than those with the highest incomes [55]. Therefore, these interaction effects suggest that a greater accumulation of NSC may help reduce socioeconomic inequality in depressive symptoms. We have also observed that the negative linkage between neighborhood participation and depressive symptoms was stronger in people who are in the unemployed group than in the employed group. One possible explanation is that people who are unemployed have more time in the neighborhood, and leisure activities and volunteering activities help them to alleviate anxiety [56]. 

These findings suggest the following implications regarding NSC interventions towards depressive symptoms in urban Chinese neighborhoods. First, our findings suggest that residents are in a better mental health position if they live in neighborhoods with a higher level of social capital. Thus, neighborhood governors, social workers, and organization leaders should act jointly to build up trust, reciprocity, and cooperation in the living spaces, and develop volunteering and leisure activities. Second, the marginal groups (people who are of lower socioeconomic class, people who are older, and people who are unemployed) should receive more attention in such interventions. Our results suggest that lower levels of NSC will aggravate socioeconomic inequalities in depressive symptoms, and increase the risks of mental health for people who are older and people who are unemployed. Thus, rebuilding the neighborhood to increase NSC is an important policy implication for an aging society with a continuous rise in socioeconomic inequality [57,58]. In economic terms, it is found that specifically targeting the right group can bring their life and satisfaction substantial improvements [59], and reduce the risk of falling into poverty in view of high healthcare costs [60]. In this regard, targeting at the right group will eventually help mitigate social inequalities [61]. Therefore, policymakers and urban planners are advised to create a more friendly environment for the marginal groups to encourage their interaction and outdoor activities. Promoting social activities (especially volunteering engagement), increasing facilities provision (e.g., sport and entertainment facilities), and the supply of neighborhood communal space (e.g., gardens and residential greenness) should be effective policies for impoverished neighborhoods. 

Some limitations to this study should be acknowledged. First, the survey upon which this study was based was conducted from 2012 to 2013, and is somewhat dated. In recent years, the development of communication devices and online technology may have affected the correlation between NSC and depressive symptoms. Nonetheless, this study provides a comprehensive and systematic framework for future studies on the relationship between mental health and neighborhood relationships in China. Second, besides the main kinds of neighborhoods considered in this study, informal housing estates such as old reform housing and urban villages, should be considered in future research. Lastly, this study was based on cross-sectional data, and was not able to infer the causal relationship between NSC and depressive symptoms. Thus, longitudinal studies are needed in future studies to comprehensively reveal the casual relationships between NSC and depressive symptoms.

## 6. Conclusions

There has been growing research interest in community building in China [42]. However, the effects of these spatial and social changes on residents’ mental health have been insufficiently explored. Drawing data from a city-wide survey in Guangzhou, China, the present study explored the relationship between neighborhood-based social capital and depressive symptoms among Chinese adults. The findings of our study not only report new evidence about the consistent beneficial effects of NSC beyond national borders in the Chinese context, but also, more importantly, highlight that NSC is especially important for the marginal population’s depressive symptoms. Particularly, our analysis confirmed that perceptions of higher levels of NCSC were negatively associated with depressive symptoms, consistent with what has been found in developed countries. In addition, the NSSC (e.g., neighborhood-based social networks, and participation in leisure, volunteering, and welfare activities) was also linked with having fewer depressive symptoms. Moreover, our analysis found that the relationships between NSC and depressive symptoms were significantly heterogeneous across socioeconomic groups. This indicates that lower levels of NSC among disadvantaged groups may aggravate depression inequalities across social classes. In addition, the association between social participation and depressive symptoms was accentuated for people who are older, and for people who are unemployed. The present study provided evidence to suggesting that NSC plays a significant role in reducing depression for people who are older who spent more time in the neighborhood, and for individuals in a lower socioeconomic class and people who are unemployed who have smaller networks outside of the neighborhood, and who have become neighborhood-dependent [54]. Thus, our study highlights the need for building neighborhoods, especially for the marginal groups. 

## Figures and Tables

**Table 1:** Principal component analysis of NCSC.

Statements	‘Neutral’ (%)	‘Agree’ (%)	‘Strongly Agree’ (%)	Mean	Component
					1
(a) People in this neighborhood can be trusted	36.33	48.72	4.56	3.47	0.697
(b) People are willing to help one another in the neighborhood	28.75	57.16	6.10	3.61	0.796
(c) I can rely on my neighbors to help with things such as collecting mail, newspaper, milk, and so on	27.11	49.06	7.59	3.46	0.618
(d) People in the neighborhood can get together to address neighborhood issues	27.57	53.91	8.99	3.61	0.735
(e) People in this neighborhood can get along with one another	13.91	73.23	10.25	3.90	0.694
(f)This is a cohesive community	32.46	48.95	7.21	3.51	0.795

**Table 2:** Sample profile (N = 1771).

	Mean	S.D.	Min.	Max.
Age	45.17	14.66	20.00	86.00
Male	0.45	0.49	0.00	1.00
Married	0.81	0.38	0.00	1.00
Years of education	12.93	3.45	6.00	21.00
Lower class	0.58	0.49	0.00	1.00
Unemployed	0.07	0.27	0.00	1.00
Child in house	0.61	0.49	0.00	1.00
Local *Hukou*	0.72	0.45	0.00	1.00
Homeownership	0.79	0.40	0.00	1.00
Years of residence	7.30	5.42	0.00	50.00
NCSC	0.00	1.00	−4.74	2.65
Discussion network	5.27	10.99	0.00	100.00
Social participation	0.81	0.35	0.00	1.00
Depressive symptoms	9.98	6.75	0.00	44.00

**Table 3:** Relationship between NSC and depressive symptoms.

	Model 1		Model 2		Model 3	
Variable	Coef.	S.E.	Coef.	S.E.	Coef.	S.E.
Age	−0.008	0.016	−0.010	0.016		
Elderly (Ref. The younger)					6.192	1.748
Gender (1 = male)	0.316	0.396	0.448	0.397	0.416	0.396
Marital status (1 = married)	−0.567	0.553	−0.526	0.551	−0.650	0.546
Years of education	0.042	0.069	0.027	0.069	0.045	0.066
Social-economic status (Ref. Middle class or above)						
Lower class	1.334 **	0.400	1.589 *	1.463	1.189 *	1.458
Unemployed	3.499 ***	1.117	15.146 ***	4.634	16.553 ***	4.619
Child						
*Hukou* (1 = local)	−0.117	0.470	−0.137	0.470	−0.225	0.464
Homeownership	−0.080	0.563	−0.036	0.565	−0.078	0.558
Years of residence	0.003	0.040	0.001	0.040	−0.004	0.039
NCSC	−0.963 ***	0.209	−1.270 ***	0.279	−1.107 ***	0.298
Discussion network	−1.176 **	0.467	−1.769 **	0.597	−1.453 *	0.645
Social participation	−2.043 ***	0.687	−1.421 *	0.855	−0.049	0.934
Lower class × NCSC			−0.597 **	0.422	−0.530 *	0.420
Lower class × Discussion network			−1.497 *	0.926	−1.561 *	0.920
Lower class × Social participation			−0.892	1.450	−1.330	1.446
Unemployed × NCSC			−0.669	1.060	−0.552	1.053
Unemployed × Discussion network			−0.093	2.981	−0.416	2.966
Unemployed × Social participation			−12.301 ***	4.730	−13.509 ***	4.711
Elderly × NCSC					−0.580	0.518
Elderly × Discussion network					−1.363 *	1.065
Elderly × Social participation					−5.616 ***	1.697
Constant	11.584	1.459	11.584	1.531	11.463	1.299
F	6.858 ***		5.371 ***		5.460 ***	
Adj_R^2^	0.063		0.071		0.081	

Note: *, **, and *** indicate statistical significance at the 5%, 1%, and 0.1% levels respectively.

## Data Availability

Data that support the findings of this study are available from the Corresponding author upon reasonable request.
